# 全自动固相萃取-超高效液相色谱-串联质谱法同时测定饮用水中全氟化合物、抗生素和农药

**DOI:** 10.3724/SP.J.1123.2025.08023

**Published:** 2026-05-08

**Authors:** Jing LIANG, Jiali FENG, Dong ZENG, Liping ZHOU, Xuan ZHONG, Yi LI, Dongyang CHEN

**Affiliations:** 湖南省疾病预防控制中心（湖南省预防医学科学院），湖南 长沙 410153; Hunan Provincial Center for Disease Control and Prevention （Hunan Academy of Preventive Medicine），Changsha 410153，China

**Keywords:** 全氟化合物, 抗生素, 农药, 超高效液相色谱-串联质谱, 固相萃取, 水, perfluorinated compounds （PFCs）, antibiotics, pesticides, ultra-performance liquid chromatography-tandem mass spectrometry （UPLC-MS/MS）, solid phase extraction （SPE）, water

## Abstract

饮用水中新污染物因其生物毒性和环境持久性，对公共健康和生态系统构成潜在威胁，亟需发展高效的监测与防治技术。目前饮用水中新污染物的检测技术主要侧重于对单一类型污染物进行分析，这不仅导致检测成本增加，还无法快速应对水质监测中多种类污染物检测的需求。本研究建立了一种新型的分析方法，基于全自动固相萃取-超高效液相色谱-串联质谱法同时检测饮用水中30种不同特性的污染物，包括全氟化合物、抗生素和农药。采用全自动固相萃取仪对水样进行自动化前处理，经OASIS HLB固相萃取柱富集净化。待测物采用ACQUITY UPLC HSS T3色谱柱（100 mm×2.1 mm，1.7 μm）进行分离，流动相为5 mmol/L乙酸铵水溶液（含0.1%甲酸）-乙腈体系，梯度洗脱，采用电喷雾离子源正/负切换模式分析测定，外标法定量。结果表明，30种化合物在0.10~200.0 μg/L范围内线性关系良好，相关系数（*r*）大于0.990，检出限为0.01~1.0 ng/L，定量限为0.03~3.0 ng/L，在不同添加水平下（0.20、1.0、10.0 μg/L），回收率为70.2%~120.0%，相对标准偏差（RSD，*n*=6）为3.2%~9.6%。采用该方法对湖南省9个县区的水样进行检测，检出16种化合物，检出含量为0.1~9.9 ng/L，其中检出率最高的是莠去津，检出含量最高的是氯虫苯甲酰胺。根据GB 5749-2022《生活饮用水卫生标准》对饮用水的管控要求，全氟辛酸、2，4-二氯苯氧乙酸、莠去津、克百威和灭草松的含量远低于国家的限值要求，表明在所研究地区的水源中这些化合物的健康风险较低。该方法高效、快速、成本低，适用于饮用水中全氟化合物、抗生素和农药的同时测定，有望为饮用水中新污染的管控和治理提供有力的技术支撑。

## Abstract (en)

Emerging organic contaminants in drinking water, characterized by biological toxicity and environmental persistence. Developing effective strategies for monitoring and controlling these contaminants in drinking water has become an urgent need to ensure drinking water safety. However， the current detection technologies for emerging organic contaminants in drinking water mainly focus on analyzing a single type of pollutant， which increases in detection costs and fails to meet the urgent demand for rapid， multi-analyte screening in water quality monitoring. This study established a novel analytical method， which was based on the automatic solid phase extraction coupled with ultra-performance liquid chromatography-tandem mass spectrometry method （SPE-UPLC-MS/MS）， enabling the simultaneous detection of 30 contaminants with diverse physicochemical properties in drinking water. The 30 contaminants included perfluorinated compounds （PFCs）， antibiotics and pesticides. The water samples were pretreated automatically by an automatic solid phase extraction instrument， and then purified and enriched through an OASIS HLB solid phase extraction column. The 30 contaminants were separated on an ACQUITY UPLC HSS T3 column （100 mm×2.1 mm， 1.7 μm） with a gradient mobile phase consisting of 5 mmol/L ammonium acetate （containing 0.1% formic acid） and acetonitrile. The samples were analyzed and determined by the positive/negative switching mode of the electrospray ionization source， and quantified by the external standard method. The results showed that the 30 compounds had good linear relationships within the mass concentration ranges of 0.10-200.0 μg/L， and the correlation coefficients （*r*） greater than 0.990. The limits of detection were 0.01-1.0 ng/L， and the limits of quantification were 0.03-3.0 ng/L. The 30 compounds exhibited recoveries in the range of 70.2%-120.0% at three levels （0.20， 1.0， 10.0 μg/L）， and the relative standard deviations （RSDs， *n*=6） were 3.2%-9.6%. Water samples from nine counties and districts in Hunan Province were analyzed using the developed method. The 16 compounds were detected， with the mass concentration range of 0.1-9.9 ng/L. Among these， atrazine was the most frequently detected， whereas the highest concentration observed was chlorantraniliprole. According to the control requirements specified in GB 5749-2022 *Standards for Drinking Water Quality*， the contents of perfluorooctanoic acid， 2，4-dichlorophenoxyacetic acid， atrazine， carbofuran， and bentazone were significantly lower than the national standard limits， indicating a low health risk associated with these compounds in the source water of the studied regions. This method is efficient， rapid， and cost-effective， which is suitable for the simultaneous determination of PFCs， antibiotics， and pesticides in drinking water. This method provides strong technical support for the control and treatment of new contaminants in drinking water.

饮用水安全是世界公共卫生领域高度关注的焦点。随着工业化进程加速，工业污水排放导致的水体有机污染日益严峻，由其引发的公共卫生事件及其对人体健康造成的威胁日益增加^［1，2］^，尤其是近年来饮用水中的新污染物引起民众的广泛关注^［3，4］^。新污染物因具有生物毒性、环境持久性、生物累积性等特点而可能危害生态环境或威胁人体健康^［5-9］^，在国务院办公厅印发的《新污染物治理行动方案》中提出“筛、评、控”的工作思路，要求以高关注、高产（用）量、高环境检出率的化学物质为重点，完成环境风险筛查和评估，动态发布重点管控新污染物清单。全氟化合物、抗生素和农药是其中关注的优先管控对象^［2，10］^，被列入我国《重点管控新污染物清单（2023版）》。然而，全氟化合物（PFCs）、抗生素和农药这3种化合物因在化学键合特性、空间结构和官能团组成上的差异导致其有不同的物理化学性质^［11，12］^。这种理化性质多样的污染物使传统的单步预处理和分析方法难以实现同时分析检测。因此，亟待开发一种高效、快速的前处理和分析方法来有效地检测这些性质迥异的污染物。

当前，国内外研究对水质中的全氟化合物、抗生素和农药的检测通常采用分类测定，如Schaefer等^［13］^采用高效液相色谱-四极杆飞行时间质谱法测定美国污水处理厂水中全氟化合物，共鉴定出37种组分，检出率为93.5%；钟蕾等^［14］^采用HC-C18固相萃取柱对水样进行富集，通过高效液相色谱-串联质谱法测定水中14种苯脲类除草剂及苯甲酰脲类杀虫剂；王锦等^［15］^采用高效液相色谱-串联质谱对环境水样中22种抗生素进行测定，地表水、污水处理厂废水口和畜禽养殖废水中有不同含量的抗生素检出。我国《生活饮用水卫生标准检验方法》（GB/T 5750-2023）也对全氟化合物、抗生素和农药提供了基础分析方法。但目前水中常发现多类污染物同时存在的情况，针对单一类别污染物的检测方法已无法快速应对日益增长的水质监测任务需求，且检测成本高。针对饮用水中全氟化合物、抗生素、农药等理化性质迥异的多类别痕量污染物的高通量检测方法，尤其结合自动化前处理的方案罕见报道。

本研究旨在开发一种适用于饮用水中全氟化合物、抗生素和农药这3类不同特性污染物的自动化前处理方法，显著提升样品处理效率和可靠性；同时基于超高效液相色谱-串联质谱（UPLC-MS/MS）平台，建立一种高效、灵敏、可靠的分析方法，实现30种理化性质差异显著的多类别污染物在饮用水中的同时准确定量与筛查。

## 1 实验部分

### 1.1 仪器和试剂

ACQUITY UPLC-XEVO-TQ-S超高效液相色谱-串联质谱仪（美国Waters公司）；SPEVA全自动固相萃取仪（睿科集团（厦门）股份有限公司）；OASIS HLB SPE柱（500 mg/6 mL，美国Waters公司）；CNWBOND HC-C18 SPE柱（500 mg/6 mL，上海安谱实验科技股份有限公司）；ENV+SPE柱（500 mg/6 mL，瑞典Biotage公司）；玻璃纤维滤膜（孔径为0.45 μm）。

全氟癸酸（PFDA）、全氟壬酸（PFNA）、全氟辛酸（PFOA）、全氟己烷磺酸（PFHxS）、全氟庚酸（PFHpA）、全氟己酸（PFHxA）、全氟丁烷磺酸（PFBS）、全氟十二酸（PFDoDA）、全氟癸烷磺酸（PFDS）、全氟十一酸（PFUnDA）、全氟辛烷磺酸（PFOS）、全氟十六酸（PFHxDA）、全氟十四酸（PFTeDA）和全氟十三酸（PFTrDA）标准溶液（质量浓度100 mg/L，溶剂是甲醇，天津阿尔塔科技有限公司）；甲氧隆、杀铃脲、氯虫苯甲酰胺、氟苯脲、氟啶脲、氟丙氧脲和氟虫脲标准溶液（质量浓度100 mg/L，溶剂是乙腈，天津阿尔塔科技有限公司）；灭草松、2，4-D、克百威和莠去津标准溶液（质量浓度100 mg/L，农业部环境保护科研监测所）；甲萘威标准溶液（质量浓度1 000 mg/L，上海安谱璀世标准技术服务有限公司）；沙氟沙星、达氟沙星、洛美沙星、培氟沙星标准品（Dr. Ehrenstorfer公司）；乙酸铵（色谱纯，上海阿拉丁生化科技股份有限公司）；甲酸（色谱纯，上海安谱实验科技股份有限公司）；甲醇、乙腈（色谱纯，德国Merck公司）；超纯水为Milli-Q纯水系统制备（美国Millipore公司）。

### 1.2 标准溶液的配制

购买的标准溶液直接使用，固体标准品则分别称取10.0 mg（精确到0.1 mg）于10 mL容量瓶中，使用超纯水溶解并定容，配制成1 000 mg/L的标准溶液。分别移取一定体积的全氟化合物、抗生素和农药标准溶液于10 mL容量瓶中，甲醇定容至刻度，配成每种化合物的质量浓度为1.0 mg/L的混合标准储备液。临用前，将上述混合标准储备液用甲醇稀释成质量浓度为0.1、0.2、0.5、1.0、5.0、20.0、50.0、100.0、200.0 μg/L的系列标准工作液。

### 1.3 样品前处理

全自动固相萃取系统能自动完成固相萃取小柱的活化、样品上样、淋洗、洗脱、氮气干燥、浓缩等操作。具有高通量（连续运行64个样品）、高效、省时省力等特点。本文使用全自动固相萃取系统对水样进行前处理。

水样经玻璃纤维过滤器过滤，将OASIS HLB固相萃取小柱放入全自动固相萃取仪中，依次用6 mL甲醇和6 mL纯水活化平衡固相萃取柱，取500 mL水样以5 mL/min的流速通过固相萃取柱，用10 mL纯水淋洗固相萃取柱，在负压下氮气干燥15 min，用3 mL甲醇以2 mL/min的速度洗脱两次，洗脱液收集在15 mL离心管中，在30 ℃水浴下氮气吹至近干，然后用0.5 mL 甲醇对样品进行复溶，涡旋振荡器充分混匀后超声1 min，离心20 min，转移至进样小瓶中，用于UPLC-MS/MS分析。

### 1.4 仪器条件

#### 1.4.1 色谱条件

色谱柱：ACQUITY UPLC HSS T3色谱柱（100 mm×2.1 mm，1.7 μm）；流动相：5 mmol/L乙酸铵水溶液（含0.1%甲酸）（流动相A）和乙腈（流动相B）；梯度洗脱程序：0～1.0 min，5%B；1.0～2.0 min，5%B~40%B；2.0～3.0 min，40%B~70%B；3.0～5.0 min，70%B~90%B；5.0～6.0 min，90%B~95%B；6.0～8.0 min，95%B；8.0～10.0 min，95%B~40%B；10.0～11.0 min，40%B~5%B；11.0～12.0 min，5%B。流速：0.3 mL/min；进样量：5 μL；柱温：40 ℃。

#### 1.4.2 质谱条件

离子源：电喷雾离子源（electron spray ionization， ESI），正/负切换模式扫描（ESI^+^和ESI^-^）；负离子扫描模式下毛细管电压3.5 kV，正离子扫描模式下毛细管电压0.5 kV；脱溶剂温度：500 ℃；脱溶剂气流速：1 000 L/h；采用多反应监测（MRM）模式进行质谱检测。30种化合物的质荷比（*m/z*）、碰撞能（CE）和锥电压（CV）等参数见表1。

**表1:** 30种化合物的MRM质谱参数

No.	Compound	Abbreviation	ESI	Parent ion （*m/z*）	Product ions （*m/z*）	CEs/eV	CV/V
1	carbaryl （甲奈威）		+	202.1	145.0^*^， 127.0	10， 25	30
2	atrazine （莠去津）		+	216.1	174.0^*^， 132.0	25， 18	30
3	bentazone （灭草松）		-	239.2	132.1^*^， 197.2	20， 20	30
4	2，4-dichlorophenoxyacetic acid （2，4-二氯苯氧乙酸）	2，4-D	-	218.8	161.0^*^， 125.0	10， 18	30
5	carbofuran （克百威）		+	222.1	165.1^*^， 123.0	12， 12	20
6	sarafloxacin （沙拉沙星）		+	386.2	342.3^*^， 299.0	25， 38	30
7	danofloxacin （达氟沙星）		+	358.3	340.2^*^， 314.0	26，18	30
8	lomefloxacin （洛美沙星）		+	352.0	265.2^*^， 308.1	32， 23	30
9	pefloxacin （培氟沙星）		+	334.2	290.1^*^， 316.1	29， 25	30
10	metoxuron （甲氧隆）		+	229.0	72.0^*^， 156.0	28， 20	30
11	triflumuron （杀铃脲）		-	357.1	176.0^*^， 153.9	30， 27	30
12	chlorantraniliprole （氯虫苯甲酰胺）		-	482.0	204.0^*^， 202.0	20， 27	30
13	teflubenzuron （氟苯脲）		-	379.1	195.9^*^， 358.8	29， 9	30
14	chlorfluazuron （氟啶脲）		-	539.9	520.1^*^， 356.6	15， 28	30
15	lufenuron （氟丙氧脲）		-	509.1	325.9^*^， 175.0	27， 50	30
16	flufurazone （氟虫脲）		-	487.0	467.0^*^， 156.3	9， 21	30
17	perfluorodecanoic acid （全氟癸酸）	PFDA	-	513.0	469.0^*^， 219.0	18， 18	30
18	perfluorononanoic acid （全氟壬酸）	PFNA	-	463.0	419.0^*^， 219.0	16， 14	30
19	perfluorooctanoic acid （全氟辛酸）	PFOA	-	413.0	369.0^*^， 169.0	12， 24	30
20	perfluoroheptanoic acid （全氟庚酸）	PFHpA	-	363.0	319.0^*^， 169.0	14， 24	30
21	perfluorohexanoic acid （全氟己酸）	PFHxA	-	312.9	269.0^*^， 119.0	10， 20	14
22	perfluorobutanesulfonic acid （全氟丁烷磺酸）	PFBS	-	299.0	80.0^*^， 99.0	30， 28	30
23	perfluorohexanesulfonic acid （全氟己烷磺酸）	PFHxS	-	398.8	79.8^*^， 98.9	38， 34	30
24	perfluorodecanesulfonic acid sodium （全氟癸烷磺酸）	PFDS	-	599.0	80.0^*^， 99.0	30， 28	30
25	perfluorooctane sulfonic acid （全氟辛烷磺酸）	PFOS	-	499.0	99.0^*^， 80.0	28， 30	30
26	perfluoroundecanoic acid （全氟十一酸）	PFUnDA	-	563.0	519.0^*^， 319.0	16， 28	30
27	perfluorododecanoic acid （全氟十二酸）	PFDoDA	-	613.0	569.0^*^， 169.0	18， 36	30
28	perfluorotridecanoic acid （全氟十三酸）	PFTrDA	-	633.0	619.0^*^， 169.0	20， 38	30
29	perfluorotetradecanoic acid （全氟十四酸）	PFTeDA	-	713.0	669.0^*^， 169.0	20， 38	30
30	perfluorohexadecanoic acid （全氟十六酸）	PFHxDA	-	813.0	769.0^*^， 169.0	18， 30	30

* Quantitative ion.

## 2 结果与讨论

### 2.1 UPLC-MS/MS条件的优化

#### 2.1.1 质谱条件的优化

喹诺酮类抗生素本身具有的氨基使得其易于形成［M+H］^+^的正离子；苯脲类及苯甲酰脲类农药含有脲基及强吸电子基团，易形成［M-H］^-^的负离子；全氟化合物具有羧基或磺酸基，容易失去质子带负电荷，所以选择ESI正/负切换电离模式。使用直接灌注1 μg/mL标准溶液方式优化化合物的毛细管电压、脱溶剂温度、脱溶剂气流速、锥孔电压、碰撞电压，根据碎片离子的响应强度来选择定量离子和定性离子。当毛细管电压、脱溶剂气流速、脱溶剂温度过低时会降低电离效率以及会有仪器污染的风险，而过高容易损坏仪器。综合考虑后选择的最佳质谱参数见1.4.2节。

#### 2.1.2 色谱柱的优化

分别考察了ACQUITY UPLC HSS T3（100 mm×2.1 mm，1.7 μm）、ACQUITY UPLC BEH C18（100 mm×2.1 mm，1.7 μm）、CORTECS C18+（100 mm×2.1 mm，1.6 μm）和ACQUITY UPLC HSS PFP（50 mm×2.1 mm，1.8 μm） 4种色谱柱对目标化合物分离效果和响应值的影响。结果见附图S1（www.chrom-China.com），ACQUITY UPLC HSS T3具有最优的响应和分离效果，且色谱峰对称性好，其次为ACQUITY UPLC BEH C18色谱柱，CORTECS C18+色谱柱中部分化合物的色谱峰拖尾，而ACQUITY UPLC HSS PFP色谱柱中大多数待测物的色谱峰拖尾，响应很低，分离效果差。后续实验选择ACQUITY UPLC HSS T3色谱柱进行色谱分离。

#### 2.1.3 流动相的优化

流动相的组成和pH等因素会显著影响化合物的响应值、峰形和色谱分离的效果。因此，考察了以甲醇-水和乙腈-水为流动相的溶剂体系对目标化合物响应强度的影响。结果表明，乙腈-水为流动相使大多数目标化合物都显示出较好的响应强度。此外，研究了流动相添加剂对目标化合物的检测灵敏度和色谱分离的影响，在水相中分别加入不同体积分数（0.05%、0.1%、2%）的甲酸和不同浓度（2、5、10 mmol/L）的乙酸铵。甲酸能促进［M+H］^+^离子的形成并改善峰形，乙酸铵维持溶液的pH稳定。结果见附图S2和S3，当水相中含有5 mmol/L乙酸铵和0.1%甲酸时，所有目标化合物的峰形良好，信号响应强度和色谱分离效率均有所增强。因此，后续实验中选择5 mmol/L乙酸铵水溶液（含0.1%甲酸）-乙腈作为流动相。优化条件后30种化合物的总离子流图见图1。

**图1:**
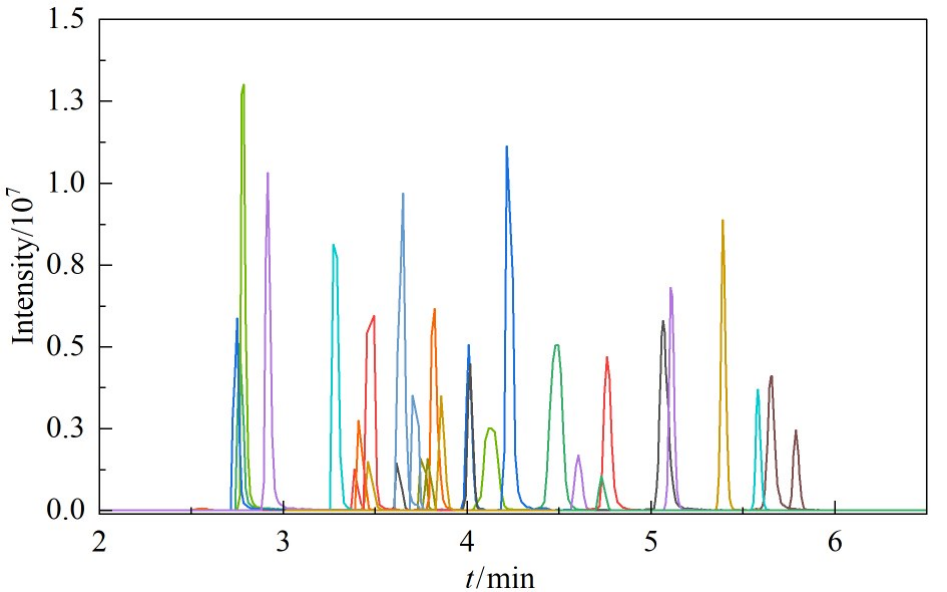
30种化合物（100.0 μg/L）的总离子流图

### 2.2 前处理条件的优化

#### 2.2.1 固相萃取柱的优化

由于分析物极性跨度大，选择对所有化合物具有可接受回收率的固相萃取柱在方法开发中极其重要。分别考察了CNWBOND HC-C18 SPE柱（500 mg/6 mL）、OASIS HLB SPE柱（500 mg/6 mL）和ENV+ SPE柱（500 mg/6 mL）对目标化合物回收率的影响。结果如附图S4所示，使用HC-C18 SPE柱时，化合物的回收率为2%~112%，其中喹诺酮类抗生素和全氟化合物的回收率小于70%，由于喹诺酮类抗生素和全氟化合物的磺酸根和羧酸根负离子会与HC-C18表面的硅羟基发生静电排斥，无法很好地保留，其回收率不好；使用ENV+ SPE柱时，化合物的回收率为0.2%~129%，其中农药的回收率很差，ENV+的填料为高度交联的聚苯乙烯-二乙烯基苯聚合物，农药主要通过与填料的氢键作用和*π-π*作用被保留，但这种作用强度很弱，致使其保留不佳，回收率低；HLB的填料是二乙烯基苯和*N*-乙烯基吡咯烷酮的共聚物，二乙烯基苯提供疏水作用位点，吡咯烷酮基团是强亲水基团，HLB可以通过反相疏水作用和氢键作用对化合物进行保留，经OASIS HLB SPE柱富集净化时，所有分析物的回收率为70.2%~120.0%。因此，选择OASIS HLB SPE柱对水样进行富集净化。

#### 2.2.2 洗脱溶剂的优化

由于不同种类的分析物在有机相与水相中的溶解度不同，选择一种合适的溶剂对所有分析物具有良好的萃取效率非常重要。本实验分别考察了甲醇、80%甲醇水溶液、50%甲醇水溶液、10%甲醇水溶液对目标分析物萃取效率的影响。结果如附图S5所示，当甲醇为洗脱溶剂时，所有分析物的回收率均大于70%，随着甲醇比例的降低，长链全氟化合物、苯脲类杀虫剂等化合物的回收率逐渐降低。由于长链全氟化合物通过极强疏水作用被保留，苯脲类杀虫剂通过疏水作用和氢键作用被保留，两类化合物与填料结合得很牢固，当甲醇的体积分数降低时，洗脱强度随之降低，分析物无法被有效地洗脱，导致其回收率降低。因此，选择甲醇作为洗脱溶剂。

### 2.3 方法学性能

#### 2.3.1 基质效应（ME）

为了验证方法的可靠性，采用空白水样对化合物配制一系列质量浓度的基质混合标准溶液，通过计算基质匹配标准曲线斜率和溶剂标准曲线斜率的比值来分析ME。ME=（基质匹配标准曲线斜率-溶剂标准曲线斜率）/溶剂标准曲线斜率×100%，当|ME|<20%时，为弱基质效应，当|ME|为20%~50%时，为中等基质效应，当|ME|>50%时，为强基质效应^［16］^。结果如附图S6所示，不同的化合物在水中存在一定的基质抑制或者基质增强效应，其中有6种化合物的|ME|小于10%，无明显基质效应；24种化合物的|ME|为10%~20%，为弱基质效应。因此，采用溶剂标准曲线进行定量分析。

#### 2.3.2 检出限、定量限和线性范围

配制系列混合标准溶液，按照1.4节的方法进行检测。以化合物的质量浓度为横坐标（*x*），以其对应的峰面积为纵坐标（*y*），绘制标准曲线。仪器检出限和定量限分别以3倍信噪比和10倍信噪比来计算，根据前处理步骤，计算方法检出限（LOD）和方法定量限（LOQ）。结果显示，所有化合物在各自的质量浓度范围内线性关系良好，线性相关系数（*r*）为0.990~0.999。方法检出限为0.01~1.0 ng/L，定量限为0.03~3.0 ng/L。所有化合物的线性范围、线性方程、相关系数、检出限和定量限见表2。

**表2:** 30种化合物的线性范围、线性方程、相关系数、检出限和定量限

Compound	Linear range/（μg/L）	Linear equation	*r*	LOD/（ng/L）	LOQ/（ng/L）
Carbaryl	0.10-100.0	*y*=1392.7*x*+126.5	0.997	0.1	0.30
Atrazine	0.10-50.0	*y*=119359*x*+952.1	0.999	0.01	0.030
Bentazone	0.10-200.0	*y*=1303.2*x*+38.5	0.998	0.5	1.5
2，4-D	0.50-100.0	*y*=605.14*x*+82.2	0.999	0.8	2.4
Carbofuran	0.10-50.0	*y*=123892*x*+6276.3	0.997	0.01	0.030
Sarafloxacin	0.10-100.0	*y*=5209.8*x*-854.9	0.998	0.02	0.060
Danofloxacin	0.50-100.0	*y*=3506.329*x*-2547.4	0.992	0.1	0.30
Lomefloxacin	0.10-100.0	*y*=12655.6*x*-1385.6	0.995	0.1	0.30
Pefloxacin	0.10-100.0	*y*=7935.2*x*-1517.0	0.994	0.1	0.30
Metoxuron	0.10-100.0	*y*=68770.8*x*+28480.0	0.995	0.1	0.30
Triflumuron	0.50-200.0	*y*=186.7*x*-27.7	0.993	1	3.0
Chlorantraniliprole	0.50-200.0	*y*=825.1*x*+121.7	0.997	0.5	1.5
Teflubenzuron	0.50-200.0	*y*=505.5*x*-125.2	0.997	0.5	1.5
Chlorfluazuron	0.50-200.0	*y*=2489.7*x*-694.3	0.997	0.3	0.90
Lufenuron	0.50-200.0	*y*=1363.3*x*-601.4	0.991	0.3	0.90
Flufurazone	0.50-200.0	*y*=515.7*x*-110.6	0.990	0.7	2.1
PFDA	0.50-100.0	*y*=1157.3*x*+523.8	0.995	0.2	0.60
PFNA	0.50-100.0	*y*=1330.6*x*+594.8	0.990	0.2	0.60
PFOA	0.50-100.0	*y*=1872.6*x*+3260.8	0.995	0.2	0.60
PFHpA	0.50-100.0	*y*=377.4*x*+209.2	0.997	0.5	1.5
PFHxA	0.50-100.0	*y*=2135.1*x*+539.8	0.991	0.2	0.60
PFBS	0.50-100.0	*y*=3962.3*x*+292.5	0.999	0.2	0.60
PFHxS	0.50-100.0	*y*=11351.5*x*+1017.1	0.999	0.1	0.30
PFDS	0.50-100.0	*y*=354.4*x*-6.3	0.992	0.5	1.5
PFOS	0.50-100.0	*y*=2657.5*x*+1240.0	0.990	0.2	0.60
PFUnDA	0.50-100.0	*y*=2646.3*x*-111.0	0.999	0.2	0.60
PFDoDA	0.50-100.0	*y*=1601.9*x*-595.0	0.994	0.3	0.90
PFTrDA	0.50-100.0	*y*=797.9*x*-328.5	0.997	0.5	1.5
PFTeDA	0.50-100.0	*y*=4735.5*x*-662.2	0.997	0.2	0.60
PFHxDA	0.50-100.0	*y*=4769.8*x*-1813.0	0.992	0.2	0.60

*y*： peak area； *x*：mass concentration， μg/L.

### 2.3 3　准确度和精密度

在空白水样中加入低、中、高（0.20、1.0、10.0 μg/L）3种水平的混合标准溶液，每份水样平行处理6份进行测定，计算方法的加标回收率和精密度。结果表明，30种化合物的回收率为70.2%~120.0%，RSD为3.2%~9.6%，具体结果见表3。

**表3:** 30种化合物在不同加标水平下的回收率与相对标准偏差（*n*=6）

Compound	0.20 μg/L		1.0 μg/L		10.0 μg/L		Compound	0.20 μg/L		1.0 μg/L		10.0 μg/L	
	Rec./%	RSD/%	Rec./%	RSD/%	Rec./%	RSD/%		Rec./ %	RSD/%	Rec./ %	RSD/%	Rec./ %	RSD/%
Carbaryl	72.5	4.9	71.3	4.0	75.9	6.7	Flufurazone	76.3	9.4	80.3	8.2	78.2	7.5
Atrazine	75.2	8.1	76.7	6.7	72.4	5.4	PFDA	107.4	9.6	115.4	6.2	85.7	6.8
Bentazone	115.0	6.2	70.4	5.3	76.2	5.1	PFNA	120.0	9.1	71.4	6.0	116.1	6.7
2，4-D	120.0	8.7	80.5	6.5	77.1	6.3	PFOA	119.6	8.7	118.9	8.9	81.4	8.1
Carbofuran	105.0	5.3	76.4	4.7	73.4	6.7	PFHpA	115.8	8.5	81.7	7.4	71.1	4.9
Sarafloxacin	90.2	7.9	119.7	7.2	106.3	7.2	PFHxA	78.5	8.6	119.5	7.2	75.4	7.8
Danofloxacin	115.0	8.1	76.4	4.7	86.5	6.4	PFBS	110.5	7.6	70.4	3.4	72.6	6.3
Lomefloxacin	83.3	3.5	78.6	5.5	90.1	5.2	PFHxS	107.5	3.2	117.4	9.1	78.3	9.1
Pefloxacin	85.4	8.3	119.4	5.7	75.4	7.5	PFDS	70.3	8.4	71.5	3.6	76.7	8.5
Metoxuron	90.1	6.0	76.7	7.6	73.2	9.3	PFOS	115.7	6.3	110.2	6.5	72.8	8.4
Triflumuron	75.7	7.6	118.7	4.6	112.4	8.1	PFUnDA	118.3	6.5	75.4	5.9	113.4	7.6
Chlorantraniliprole	90.0	8.7	70.6	7.4	72.3	7.5	PFDoDA	70.2	8.1	70.5	4.7	77.4	7.9
Teflubenzuron	120.0	8.6	71.2	8.9	76.7	6.9	PFTrDA	70.8	9.5	83.5	9.2	85.6	5.7
Chlorfluazuron	74.2	9.5	73.4	7.8	112.2	8.6	PFTeDA	75.4	8.8	116.6	8.4	71.5	4.2
Lufenuron	70.8	6.9	71.7	3.6	77.5	7.4	PFHxDA	80.6	6.9	119.2	7.6	115.4	6.8

Rec.： recovery.

### 2.4 实际水样的分析

采用建立的方法对湖南省9个县区的9份水源水进行测定。结果表明，除洛美沙星、甲氧隆、杀铃脲、氟苯脲、氟啶脲、氟丙氧脲、氟虫脲、PFHpA、PFBS、PFHxS、PFDS、PFOS、PFDoDA和PFHxDA外，其余分析物均有检出。其中检出率最高的是莠去津，每份水样均有检出；检出含量最高的是氯虫苯甲酰胺，分析物的检出含量为0.1~9.9 ng/L，具体结果见表4。

**表4:** 实际水样的测定结果 (ng/L)

Compound	Sample Nos.								
	1	2	3	4	5	6	7	8	9
Carbaryl	-	-	0.2	-	-	-	-	-	-
Atrazine	1.0	0.3	9.7	1.2	0.4	2.7	0.7	2.1	0.6
Bentazone	-	-	-	-	1.0	-	-	-	-
2，4-D	3.3	5.8	-	-	1.8	-	-	1.9	-
Carbofuran	0.2	-	0.2	-	0.1	-	-	0.1	-
Sarafloxacin	-	-	-	-	-	-	0.3	-	-
Danofloxacin	-	-	-	3.9	-	-	-	-	-
Pefloxacin	-	-	-	-	-	-	2.1	2.4	2.0
Chlorantra-niliprole	8.6	5.3	2.7	9.9	4.5	1.5	8.5	-	0.8
PFDA	1.0	-	-	-	0.3	0.3	-	-	-
PFNA	-	-	0.4	-	-	0.3	0.3	-	1.4
PFOA	2.3	-	3.9	4.5	2.3	-	3.1	3.3	4.6
PFHxA	-	-	0.8	0.3	-	-	0.4	-	-
PFUnDA	-	-	0.4	-	-	-	-	-	0.3
PFTrDA	-	-	-	0.7	-	0.5	-	-	0.5
PFTeDA	-	-	-	0.6	-	-	-	-	-

-： not detected. The compounds that were not detected in all samples were not included in the table.

由GB 5749-2022 《生活饮用水卫生标准》对饮用水的管控要求可知，PFOA的限值是80 ng/L，2，4-D、莠去津、克百威和灭草松的限值分别是0.03、0.002、0.007和0.3 mg/L。本研究中分析物的含量范围为0.1~9.9 ng/L，远低于国家的限值要求，表明湖南省这9个县区的水源水中这些物质的健康风险较低。

## 3 结论

本研究建立了一种全自动固相萃取-超高效液相色谱-串联质谱法同时测定饮用水中30种新污染物的方法，解决了传统手动固相萃取对水样前处理时流速难以控制，前处理平行性差，耗时费力的问题，大大提高了实验效率。该方法为水质中全氟化合物、抗生素和农药的高效测定提供了技术支撑，同时为饮用水的健康风险评价提供了依据。
